# Comparative assessment of Graves’ disease and main extrathyroidal manifestation, Graves’ ophthalmopathy, by non-targeted metabolite profiling of blood and orbital tissue

**DOI:** 10.1038/s41598-018-27600-0

**Published:** 2018-06-18

**Authors:** Dong Yoon Ji, Se Hee Park, Soo Jin Park, Kyoung Heon Kim, Cheol Ryong Ku, Dong Yeob Shin, Jin Sook Yoon, Do Yup Lee, Eun Jig Lee

**Affiliations:** 1 The Department of Bio and Fermentation Convergence Technology, BK21 PLUS Program, Kookmin University, Seoul, Republic of Korea; 2 Division of Endocrinology and Metabolism, Department of Internal Medicine, Yonsei University College of Medicine, Seoul, Republic of Korea; 3 Graduate School, Yonsei University College of Medicine, Seoul, Republic of Korea; 4 The Department of Biotechnology, Graduate School, Korea University, Seoul, Republic of Korea; 5 Institute of Endocrine Research, Yonsei University College of Medicine, Seoul, Republic of Korea; 6 Department of Ophthalmology, Yonsei University College of Medicine, Seoul, Republic of Korea

## Abstract

Graves’ disease (GD) is an autoimmune disorder that causes the overproduction of thyroid hormones and consequent cascade of systemic metabolism dysfunction. Moreover, Graves’ ophthalmopathy (GO) is the main extrathyroidal manifestation of Graves’ disease (GD). The goal of the study was to identify metabolic signatures in association with diagnostic biomarkers of GD without GO and GO, respectively. Ninety metabolites were profiled and analyzed based on a non-targeted primary metabolite profiling from plasma samples of 21 GD patients without GO, 26 subjects with GO, and 32 healthy subjects. Multivariate statistics showed a clear discrimination between healthy controls and disease group (R2Y = 0.518, Q2 = 0.478) and suggested a biomarker panel consisting of 10 metabolites. Among them, most of metabolites showed the positive association with the levels of thyrotropin receptor antibodies. With combination of proline and 1,5-anhydroglucitol, which were identified as GO-specific modulators, the re-constructed biomarker model greatly improved the statistical power and also facilitated simultaneous discrimination among healthy control, GO, and GD without GO groups (AUC = 0.845–0.935). Finally, the comparative analysis of tissue metabolite profiles from GO patients proposed putative metabolic linkage between orbital adipose/connective tissues and the biofluidic consequences, in which fumarate, proline, phenylalanine, and glycerol were coordinately altered with the blood metabolites.

## Introduction

Graves’ disease is the most common cause of hyperthyroidism, which results from the stimulation of autoantibodies to the thyrotropin receptor of thyroid follicular cells^1^. It usually occurs between 30 and 50 years of age, but all ages can be affected^2,3^. Similar to other autoimmune diseases, Graves’ disease occurs more frequently in women than in men, with a ratio of about 5/1^4^. Graves’ ophthalmopathy is clinically presented by about 25%-50% of patients with Graves’ disease^5^. Subclinical ophthalmopathy in orbital imaging is reported in nearly 70% of patients with Graves’ disease^6^ and severe ophthalmopathy affects 3%–5% of patients, with sight-threatening complications, such as corneal breakdown or compressive optic neuritis^7^.

Graves’ disease can be diagnosed with clinical signs and symptoms of hyperthyroidism, thyroid function tests, and positivity of thyrotropin (thyroid stimulating hormone, TSH) receptor antibodies (TRAb). Although TRAb assays have relatively high sensitivity and specificity for Graves’ disease^8^, some patients show normal TRAb levels. In those cases, radionuclide scanning can be helpful for discriminating the disease from other causes of hyperthyroidism; however, the examination takes a relatively long time and is contraindicated for certain patients, such as pregnant women. Diagnosis of Graves’ ophthalmopathy is based on the examination of ophthalmologists combined with orbital imaging^9^. However, no simple diagnostic serum marker distinguishing ophthalmopathy among Graves’ disease has been developed yet.

Recently, studies on metabolomics are widely conducted to discover diagnostic biomarkers or indicators of drug responsiveness, or to elucidate underlying pathogenesis of diseases. The diagnosis of many autoimmune diseases is delayed due to their obscure symptoms, and their pathogenesis remains to be elucidated^10^. Research studies on metabolomics using various biological specimens in autoimmune diseases have been actively conducted. Therefore, this study aimed to investigate novel biomarkers using metabolomics in Graves’ disease.

Here, non-targeted metabolite profiling was performed using GC-MS to characterize the Graves’ disease and to explore the potential biomarker for clinical application. GC-MS-based metabolite profiling is known for its reproducibility, robustness, and widely available public database^11–14^, which allows the technology to be most competent for exploratory or hypothesis-generating research^14^. Accordingly, the multivariate statistical modeling with primary metabolites suggested a putative biomarker panel unique to the disease, and the dysfunctional metabolic traits were mainly characterized by the hyperactivity of the central carbon/nitrogen metabolisms. Furthermore, the metabolic discrepancy was identified in the patients with GO compared to the ones without GO and the healthy control that may aid the mechanistic understanding of the underlying pathophysiology of this disease subtype.

## Results

### Clinical characteristics

The total study subjects were 79 patients with a mean age of 35.8 ± 10.4 years. Six out of the 32 healthy subjects (18.8%) were male; 6 (28.6%) and 7 (26.9%) of the 21 GD patients without GO and 26 GO patients, respectively (p = 0.656), and this sex ratio is similar to that of Graves’ disease. The mean TSH, free T4, and T3 levels of GD patients without GO and GO group were within normal range without a statistical difference. The mean TRAb levels of both groups were not significantly different (6.32 ± 8.28vs. 11.70 ± 13.87, p = 0.140); however, the mean TSI was higher in the GO group than in the GD patients without GO (289.0 ± 139.8 vs. 462.3 ± 171.2, p = 0.003). When evaluating the thyroid status of patients, 11 (52.4%) of the GD patients without GO and 12 (46.2%) of GO patients had euthyroid. Among GD without GO group and GO group, patient with subclinical hypothyroidism were 2 (9.5%) and 2 (7.7%), respectively. Six (28.6%) and 10 (38.5%) patients of each group had subclinical hyperthyroidism. The number of patients with overt hyperthyroidism was 2 in each group. Nineteen (90.5%) patients of GD without GO group and 24 (92.3%) of GO group were taking antithyroid drugs. The proportion of patients with antithyroid drugs or levothyroxine for each group was not statistically different (Table 1). Among patients with GO, 11 patients took steroids, 3 patients received radiation therapy for treatment of ophthalmopathy before sample collection. However, no patients with GO was receiving those treatment at the time of sample collection.

**Table 1:** Baseline characteristics of patients.

Parameters	GD (n = 21)	GO (n = 26)	P value
Age, years	36.4 ± 10.4	39.5 ± 10.3	0.316
Male gender, n (%)	6 (28.6%)	7 (26.9%)	0.900
Disease duration, month	33.5 ± 32.3	39.4 ± 46.6	0.625
T3, ng/mL	1.04 ± 0.31	1.11 ± 0.37	0.474
Free T4, ng/dL	1.02 ± 0.19	1.13 ± 0.25	0.108
TSH, μIU/mL	2.13 ± 4.09	1.49 ± 3.56	0.570
TRAb, IU/L	6.32 ± 8.28	11.70 ± 13.87	0.140
TSI, %	289.0 ± 139.8	462.3 ± 171.2	0.003
Thyroid function status, n (%)			0.915
Euthyroidism	11 (52.4%)	12 (46.2%)	
Subclinical hypothyroidism	2 (9.5%)	2 (7.7%)	
Subclinical hyperthyroidism	6 (28.6%)	10 (38.5%)	
Overt hyperthyroidism	2 (9.5%)	2 (7.7%)	
Methimazole dose, mg	8.6 ± 5.1	11.8 ± 7.1	0.090
Antithyroid drug administration, n (%)	19 (90.5%)	24 (92.3%)	0.610
Levothyroxine dose, mcg	40.5 ± 47.1	52.9 ± 43.8	0.355
Levothyroixne administration, n (%)	11 (52.4%)	18 (69.2%)	0.237
CAS			
0		14 (53.8%)	
1		3 (11.5%)	
2		3 (11.5%)	
3		3 (11.5%)	
4		1 (3.8%)	
Proptosis, mm		18.6 ± 2.6	
NOSPECS		2.7 ± 1.6	
Previous treatment for GO			
Glucocorticoid		11 (42.3%)	
Radiation therapy		3 (11.5%)	

Data are presented as mean ± SD or number (%). GD, Graves’ disease; GO, Graves’ ophthalmopathy; CAS, clinical activity score.

### Quality control of mass spectrometric analysis

Prior to data analysis, we evaluated the stability and reliability of the mass spectrometric analysis. To minimize the potential of a systematic error including analytical stability, extraction process, derivatization, and mass spectrometric analysis were performed on all samples in randomized order. In addition, the quality control (QC) mixture consisting of 30 representative metabolites was analyzed every ten samples. The data for the quality control is provided as a score plot (Supplementary Fig. S1A), a score control chart (PC1 and PC2) of the QC mixture analyzed by PCA, which presented the constant levels of the compounds throughout the analysis (Supplementary Fig. S1B)^12^.

### Evaluation of confounder effects on blood metabolism

Prior to the data analysis, we inspected a deleterious effect, which originated from drug medication on the metabolome. Multivariate statistics, MANCOVA, and PCA were applied to interrogate global impact of the medication on the blood metabolome^15,16^. The treatment duration with methimazole was analyzed as a covariate, which may indicate the level of the medication, thus differentially affecting metabolite abundances. The result showed methimazole was not significantly related to blood metabolite levels in Graves’ disease group (F = 2.881, p = 0.441). Score scatter plot by PCA confirmed that no cluster was identified by the different levels of methimazole examined (Supplementary Fig. S2). Steroid and statin administrations were examined as covariates. The results indicated that their effects on the integrative blood metabolome were not generally significant (steroid: F = 1.698, p = 0.553 and statin: F = 0.577, p = 0.806). Potential effects of decreased disease activity due to a broad spectrum of disease duration and therapy were further examined at the metabolome level within the GO group. Disease duration and therapy were not significantly correlated with the disease metabolome (disease duration: F = 0.180, p = 0.973, steroid: F = 0.651, p = 0.773, MANCOVA). Univariate statistical analysis supported the results where only two metabolites were affected by the previous steroid treatment. In addition, the score scatter plots by PCA demonstrated the minimal effects of combinatorial interactions among disease severity classifications and therapy on the blood metabolome (Supplementary Fig. S3). Likewise, the metabolic profiles of orbital adipose/connective tissues presented very low level of variation, with 24% relative coefficient of variation (%CV).

### Multivariate statistical analysis and biomarker discovery of Graves’ disease

**Figure 1:**
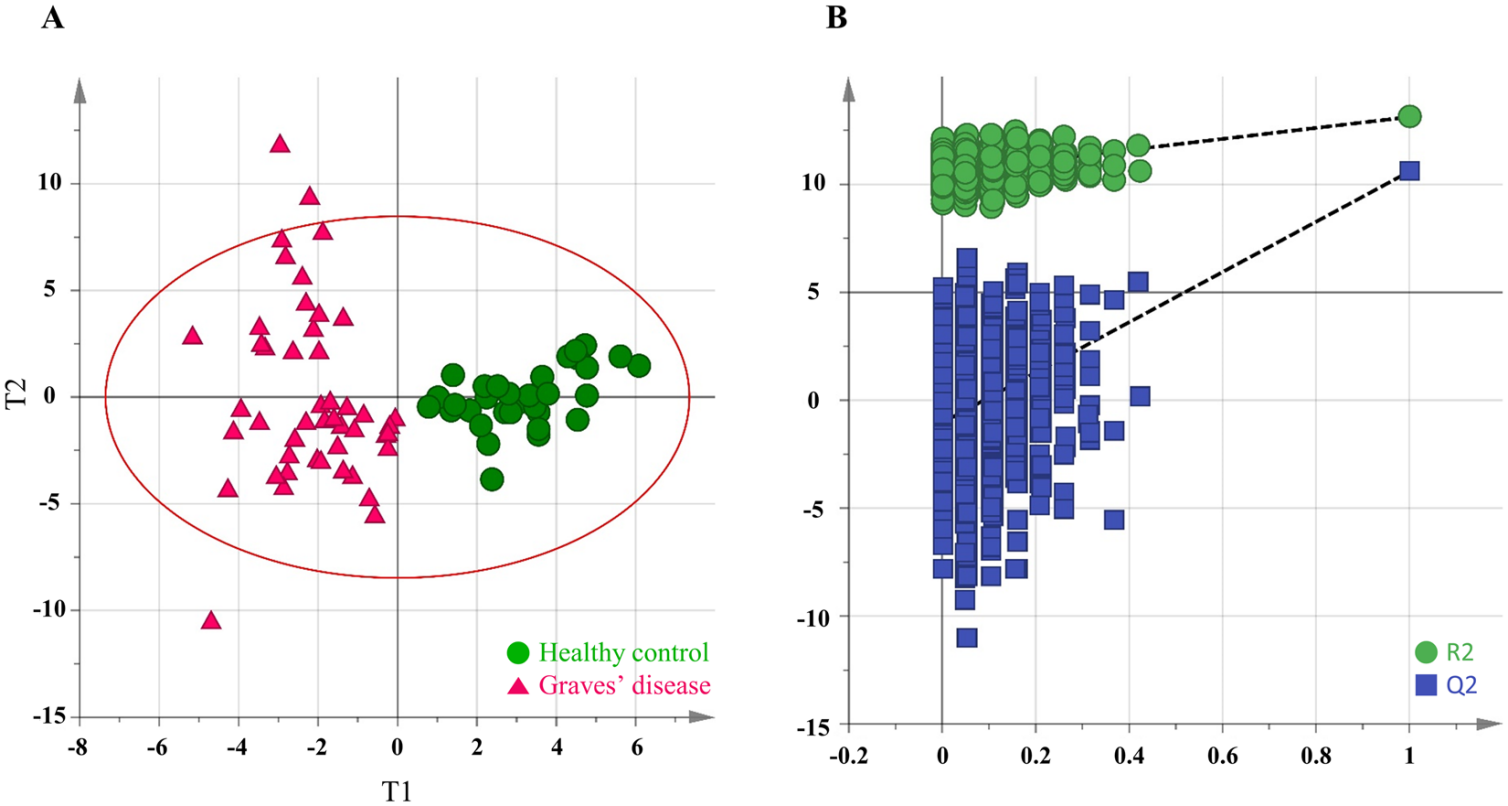
Multivariate statistical model by OPLS-DA and model validation based on permutation test. (**A**) The score plot for the first two predictive component (t[1] and t0[1]) discriminates plasma metabolite profiles between healthy control and Graves’ disease groups. Red circle is T2 ellipse indicating 0.05 as significance level. (**B**) 999 times random permutation plot with 4 components on the datasets that corresponded to the two groups (healthy controls and GD). The vertical axis corresponds to R2 (green points) and Q2 (blue points) values, presenting the goodness of fit and predictability of the original model, respectively. The horizontal axis indicates the correlation coefficient between the original Y-variable and the permuted Y-variable. The test demonstrates the OPLS-DA model’s robustness by the criteria where the original values are plotted on the right and higher than those of the 999 permuted models.

Non-targeted profiling focusing on primary metabolite was performed on a total of 79 plasma samples using GC-TOF MS. *Binbase* algorithm identified and semi-quantified 90 blood metabolites based on Fiehn library and NIST08 library^17,18^. The compounds fairly covered various biochemical categories, such as carbohydrate, fatty acid, amino acid, and organic acid. The data can be downloaded from the website (https://lms2.kookmin.ac.kr:446/index.php?hCode=PAPER_LIST&publication_name=inter_paper). To examine whether integrative blood metabolite profiles can distinguish patients with Graves’ disease from the control group, we performed unsupervised multivariate statistics, PCA, using the first two principal components. The score plot, however, did not show clustering of subjects between the disease group and control. Thus, orthogonal projection to latent structure-discriminant analysis (OPLS-DA), supervised multivariate statistics, was applied to obtain an overview of the metabolic uniqueness and potential biomarker. The OPLS-DA model performed with 7-fold cross validation showed the high levels of an explained variance (R^2^Y) of 0.812 and predictability (Q^2^Y) of 0.559 (Fig. 1A). To avoid overfitting, the model was validated with permutation test with 999 iterations that resulted in intercepts of R^2^ and Q^2^ with values of 0.555 and −0.594, respectively (Fig. 1B).

**Figure 2:**
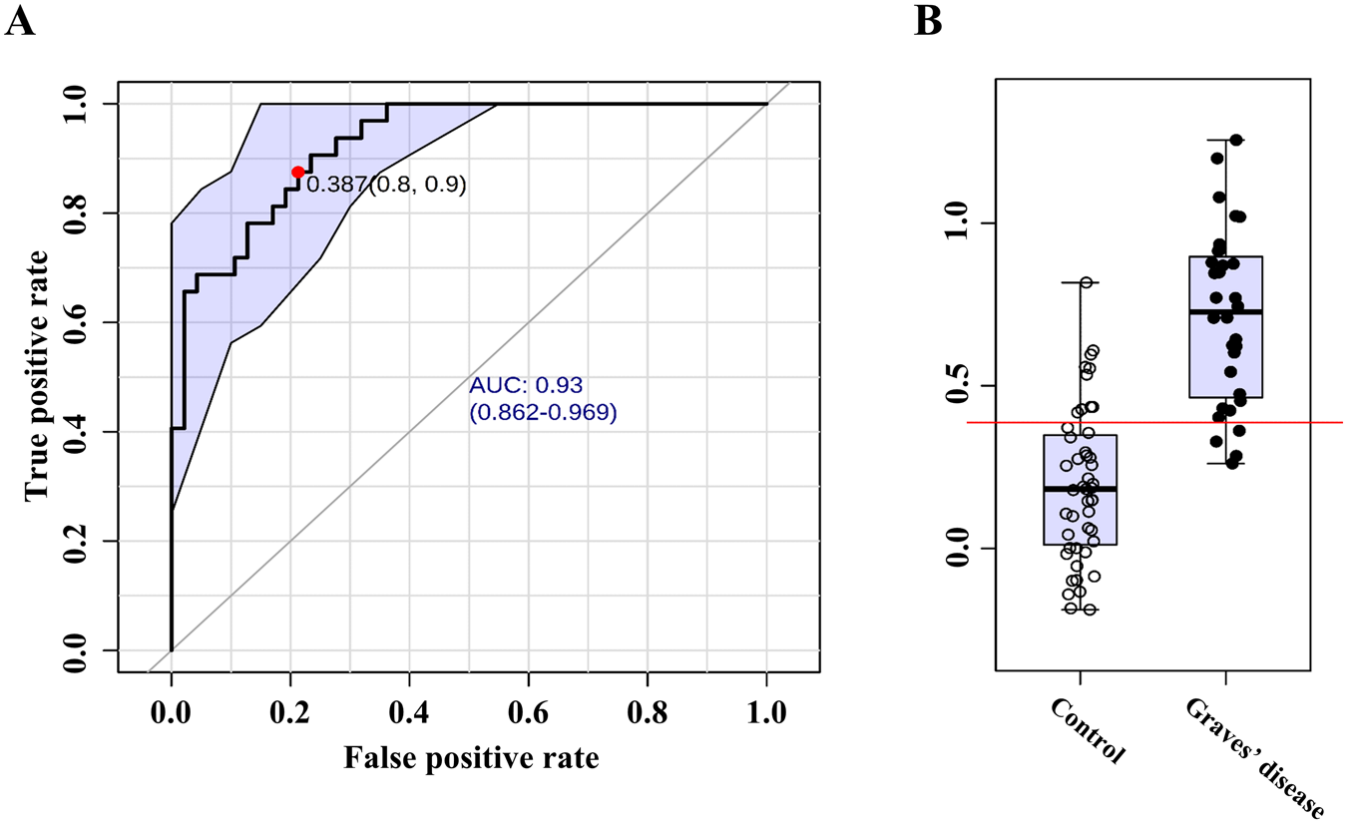
(**A**) Receiver operating characteristic (ROC) analysis of multiple metabolite panels for discriminating the healthy control and Graves’ disease group. Score matrix (t[1]) is computed based on relative concentration of 10 metabolites by OPLS-DA and the single numerical variable is introduced for ROC analysis. The 10 metabolites are glucose, pelargonic acid, 2-(4-hydroxyphenyl)ethanol, gluconic acid lactone, glycerol, pentadecanoic acid, mannose, threose, fumaric acid, and pyruvic acid. The value of the area under curve (AUC) is 0.93 (95% confidence interval: 0.862–0.969). Optimal cutoff is determined using the closest to top-left corner and the 95% confidence interval is calculated using 500 bootstrappings. (**B**) Box-and-whisker plot present the relative abundances of the variable (t[1] component) of healthy control (left) and GD (right).

Based on the model, biomarker candidates were prioritized by variable importance in project (VIP)^19^. Considering the potential applicability^20^, the number of the putative biomarkers was limited to 10 metabolites. The metabolites were glucose, pelargonic acid, fumaric acid, gluconic acid, glycerol, mannose, threose, pentadecanoic acid, pyruvate, and 2-(4-hydroxyphenyl)ethanol. Following the re-composition of the metabolite panel, the performance as biomarker was assessed using the receiver operating characteristic (ROC) curve analysis. The area under ROC curves (AUC), sensitivity, and specificity were computed with the confidence intervals for the metabolite re-composite. The AUC of the biomarker panel was 0.931 for the disease against the healthy control with 0.787 of sensitivity and 0.875 of specificity (Fig. 2). The 95% confidence intervals (CIs) were computed using non-parametric re-sampling (500-time bootstrapping). In addition, ROC analysis was performed on a data set that was randomly selected and composed of 30% (26 subjects) of all subjects (79 subjects) as a validation set. This step was repeated three times (Supplementary Fig. S4).

### A range of metabolic disturbance in primary metabolism by the disease

**Figure 3:**
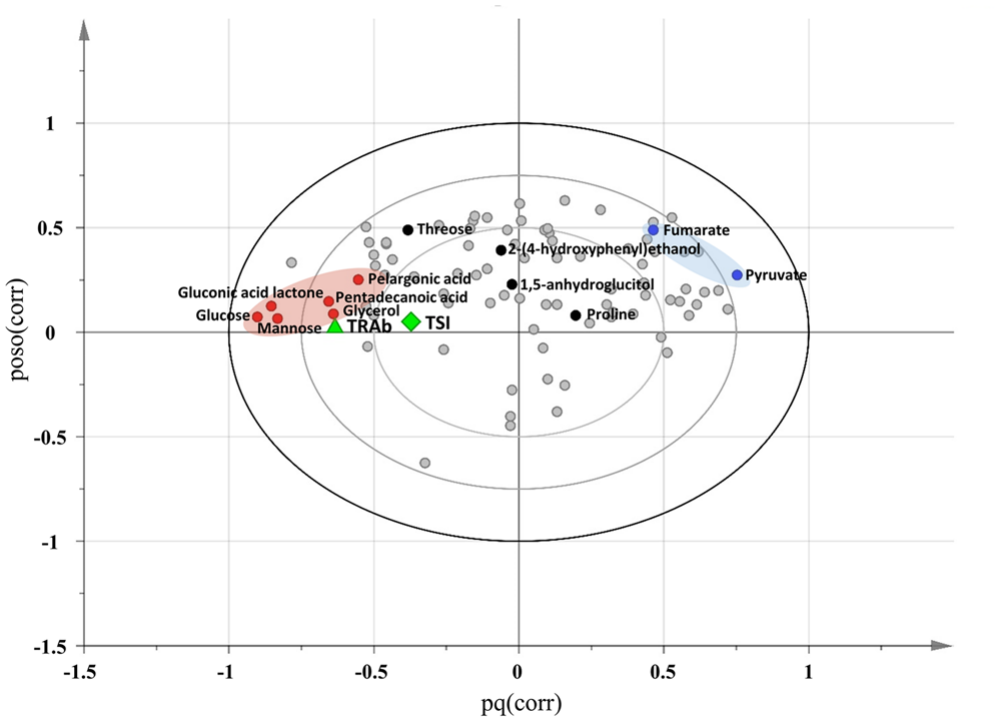
Overview of the relationship between known immune markers (TRAb and TSI) and predictive metabolites. (**A**) Variable mapping on loading scatter plot by two-way orthogonal projection to latent structures (O2PLS). Metabolite and known immune markers (TRAb and TSI) are assigned as X and Y variables, respectively. Loading of the markers and GC-TOF/MS datasets was combined to one vector. X-axis and Y-axis indicate the combined vectors, pq(corr) and poso(corr) based predictive components 1 and 2, respectively. The resultant loading plot is set to correlation scale. Distance among variables indicates the level of association. Variables in opposite direction from center present a negative relation. Only metabolite name of biomarker constituent is visualized. Pink and blue ovals indicate positively and negatively associated metabolites with TRAb.

Next, we explored a potential linkage of the molecular biochemistry of the disease reflected in the blood metabolite. Thus, we interrogated the compositional changes in metabolites. Including 10 metabolites that were selected as components of the potential biomarker panel, a total of 37 compounds were significantly different between the disease group and healthy controls (Table 2). Seventeen metabolites were significantly up-regulated, whereas 20 compounds were of lower abundance in the disease group. The most significant increase was found in pyruvate, alpha-ketoglutarate, uracil, fructose, proline, and glucose 6-phosphate. The dramatic down-regulation was observed in glucose, mannose, gluconic acid lactone, and pentadecanoic acid. Pathway enrichment analysis proposed the most significant alteration in sugar metabolism, including galactose metabolism, starch metabolism, and pentose phosphate pathway (Supplementary Fig. S5). Others were glycerolipid and amino-sugar metabolism. A range of amino acid metabolisms was characterized by a high rank of pathway impact, which implies that the key metabolites in the pathways were significantly altered (relative-betweenness centrality > 0.2). Next, we sought a potential linkage of the dysregulated metabolites with two clinical parameters that presented abnormality after medication, TRAb and TSI. Two-way OPLS (O2PLS) was applied to identify putative relation that resulted in clusters where distance and direction among variables indicated positive and negative correlations^21^. The association analysis identified the metabolites that were closely associated with TRAb whereas TSI showed comparably moderate relation to the metabolites (pq(corr) < 0.5) (Fig. 3). Most of the metabolites of the biomarker signature positively correlated with TRAb level were the constituents of the biomarker panel (glucose, mannose glycerol, pelargonic acid, and pentadecanoic acid). In addition, the strong negative correlation was identified with pyruvate. Others were 2-hydroxyhexanoic acid and 2-ketoisocaproic acid under negative association with TRAb.

**Table 2:** The list of blood plasma metabolites that present the significant differences between Graves’ disease and the healthy control.

Graves’ disease vs Control			Graves’ disease vs Control		
Metabolites	*p-*value	Fold change	Metabolites	*p-*value	Fold change^a^
Pyruvate	2.09E-05	5.21	Stearic acid	4.02E-05	0.85
Alpha-ketoglutarate	1.37E-03	2.61	1,5-anhydroglucitol	4.46E-02	0.82
Uracil	1.46E-03	1.96	Malonic acid	3.58E-03	0.82
Fructose	3.30E-02	1.75	Salicylaldehyde	2.26E-04	0.80
Proline	6.28E-03	1.70	Hexonic acid	6.43E-03	0.79
Glucose-6-phosphate	3.56E-04	1.67	1-monopalmitin	2.57E-03	0.77
Oxoproline	4.30E-03	1.57	Threose	3.27E-07	0.77
Citramalic acid	1.03E-02	1.55	Xanthine	6.74E-03	0.75
Ornithine	2.42E-03	1.42	Uric acid	3.59E-02	0.75
Fumaric acid	4.33E-05	1.40	Glycerol	5.83E-08	0.71
Adenosine-5-monophosphate	1.11E-02	1.40	Galactonic acid	2.05E-05	0.69
Fructose-6-phosphate	2.18E-02	1.30	3-hydroxypyridine	1.06E-03	0.69
2-hydroxyhexanoic acid	3.41E-03	1.29	2-(4-hydroxyphenyl)ethanol	1.09E-07	0.67
Phenylalanine	8.82E-04	1.22	2-hydroxypyridine	3.46E-04	0.64
2-ketoisocaproic acid	2.73E-02	1.21	Pelargonic acid	1.43E-07	0.63
Beta-alanine	2.70E-02	1.17	Sorbitol	1.36E-06	0.63
Valine	3.77E-02	1.13	Pentadecanoic acid	4.32E-07	0.60
			Gluconic acid lactone	1.50E-07	0.57
			Mannose	6.14E-06	0.56
			Glucose	1.55E-09	0.50

^a^Data expressed as fold to healthy control.

### Unique metabolic dysregulation of Graves’ ophthalmopathy defined by blood and tissue metabolome

**Figure 4:**
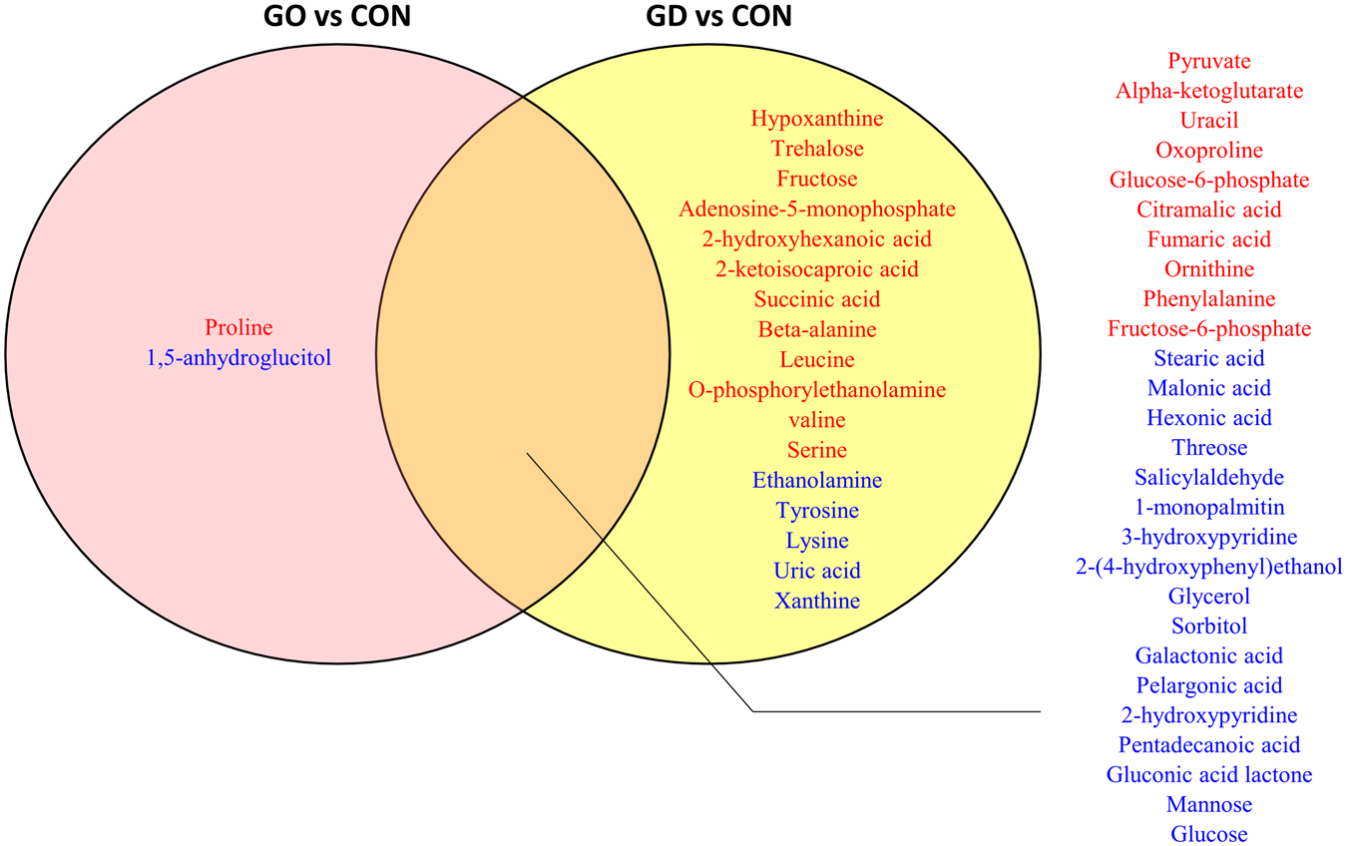
Venn diagram of the metabolite list indicating the disease type-specific and -common types. Pink and yellow circles include the metabolites that are present at significantly different levels in GD with GO and GD without GO, respectively. The overlapping region includes common metabolites that pass through statistical criteria with same direction. Red indicates the metabolites with the significantly increased levels compared to the healthy control whereas blue presents the metabolite with significantly lower levels compared to the healthy control (p < 0.05).

**Figure 5:**
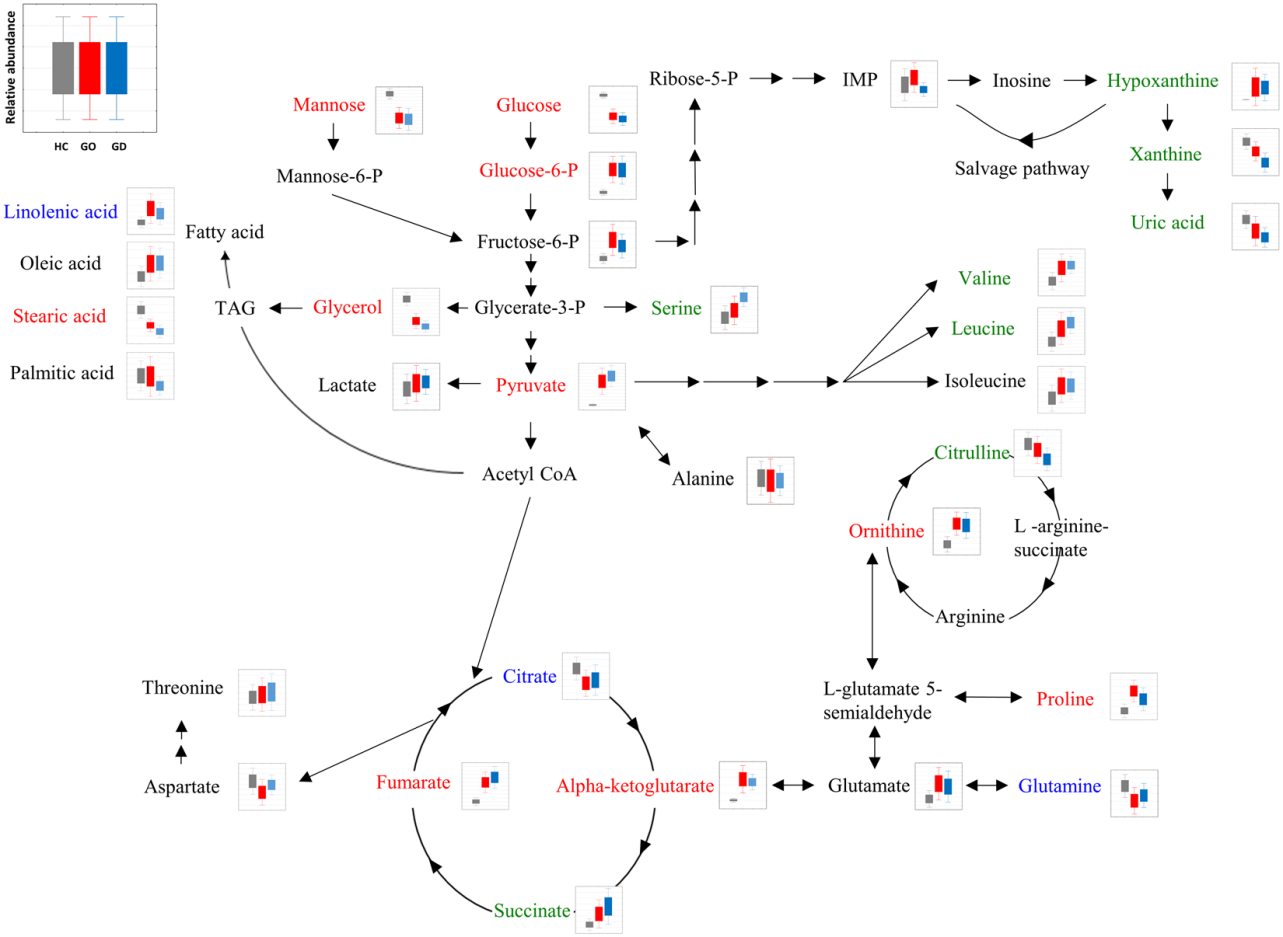
Primary metabolic pathway representing distinctive metabolic dys-regulation among GO, GD, and healthy control. Pathway is manually re-organized for visual clarification and better explanatory overview on based on metabolites that are present at different concentrations and mappable into central carbon and nitrogen metabolism. Metabolite labeled with red color shows different levels of concentration both in GC and GO compared to healthy controls. Metabolite labeled with blue color presents exclusive difference in metabolites contents between GO and the healthy control whereas green color shows the significantly different abundance only in GD compared to healthy controls (p < 0.1). The relative concentrations are present as box-and-whisker plot indicating 1 × S.E. and 1.96 × S.E., respectively.

Graves’ ophthalmopathy (GO) is an autoimmune inflammatory disorder of the orbit and periorbital tissues, characterized by upper eyelid retraction, lid lag, swelling, redness (erythema), conjunctivitis, and bulging eyes (exopthalmos)^22^. It occurs most commonly in individuals with Graves’ disease^7^. Since no blood biomarker that uniquely diagnoses Graves’ disease has been developed, we sought the unique features reflected in the patients’ blood metabolites with GO. The pair-wise comparison with the control group showed the GO-specific and GD-common metabolic signatures (Fig. 4). Most of the metabolites were commonly altered in both subtypes or only at different levels in GD. Exclusive differences in GO were found in proline and 1,5-anhydroglucitol. The direct comparison of the primary metabolites indicated the significant differences in 1,5-anhydroglucitol and ethanolamine GD and GO. The metabolite levels of GO were similar to those of GD, but some extension of specificity was identified with the relatively lower statistical criteria (p < 0.1) as summarized in Fig. 5. The metabolic intermediates of purine metabolism were present at higher levels in GO (IMP, xanthine, and uric acid). In contrast, the over-production of amino acids and TCA cycle intermediates was characteristic for the GD patients.

Subsequently, we examined if the metabolites presenting GO specificity may aid in the development of a diagnostic parameter in combination with 10 pre-selected metabolite panels that showed the moderate levels of discrimination between the patients with GO and GD. Indeed, the re-constructed biomarker model based on the 12 metabolites significantly improved the discrimination power among the three groups (healthy controls, GO, and GD without GO). The model achieved AUC values that ranged from 0.845 to 0.935 in all diagnostic sets (Supplementary Fig. S6).

Lastly, we analyzed the metabolic profiles collected from the orbital adipose/connective tissues of the GO patients and compared them with the corresponding tissues of healthy controls. A total of 24 metabolites were significantly different in the GO patients, in which 11 and 13 compounds were present at higher and lower contents, respectively, compared to the healthy control (Table 3). The up-regulated metabolites included proline, fumarate, and phenylalanine that were consistent with the expression pattern in blood metabolite analysis. The activated tissue metabolism was represented by amino acids, including asparagine, valine, allo-threonine, methionine, and glycine. Intermediates of nucleotide metabolism (adenine, guanosine, and inosine) showed the decreased levels in the tissue of GO patient that was accompanied by the reduction of ribose, glucose 6-phosphate, and fructose 6-phosphate. As to the consistency, the decreased level in glycerol was compatible with the change in blood levels.

**Table 3:** The list of tissue metabolites that present the significant differences between Graves’ ophthalmopathy (GO) and the healthy control.

Graves’ op vs Control		
Metabolites	*p-*value	Fold change^a^
Proline	1.50E-02	2.34
Malic acid	3.27E-02	2.27
Asparagine dehydrated	5.97E-04	2.24
Xanthosine	8.37E-03	2.22
Valine	1.32E-03	2.07
Fumaric acid	2.48E-02	2.05
Allothreonine	5.61E-03	2.05
Methionine	3.96E-03	1.94
Phenylalanine	6.80E-03	1.62
Asparagine	2.70E-03	1.52
Glycine	2.21E-02	1.40
Salicylaldehyde	4.66E-02	0.69
Glycerol	4.09E-02	0.69
Inositol-4-monophosphate	2.56E-02	0.64
Cholic acid	2.90E-02	0.53
Phosphoethanolamine	2.86E-02	0.48
Palmitic acid	1.82E-02	0.45
Ribose	1.93E-02	0.43
Adenine	1.63E-02	0.42
Fructose-6-phosphate	1.16E-02	0.34
Glucose-6-phosphate	3.71E-02	0.26
Guanosine	1.19E-02	0.25
Sucrose	4.00E-02	0.25
Inosine	3.69E-02	0.17

^a^Data expressed as fold to healthy control.

## Discussion

To our knowledge, the current study is the first metabolomics investigation on GO particularly at both levels of blood plasma and orbital tissue. The interrogation first demonstrated that blood metabolic profiles were unique to GD, and biomarker cluster discriminated GD from healthy controls. The metabolome-wide multivariate correlation analysis identified putative association of TRAb, a pathognomonic marker with the metabolites that were selected as the biomarker cluster. Consecutive examination on the blood plasma and orbital fibroblast tissue of the patients diagnosed as GO presented unique and consistent metabolic traits despite broad disease spectrum (therapies and disease severity).

GD is an autoimmune disorder triggered by confluence of genetic and environmental factors. Genetic studies have identified genetic susceptibility associated with GD (e.g. thyroid stimulating hormone receptor (TSHR) gene and thyroglobulin (*TG*) gene)^23,24^. A recent study has revealed the mechanistic causality, in which microenvironmental cues (e.g. cytokine) modulated chromatic structure with a single-nucleotide polymorphism (SNP)^25^. Since some metabolic features signify phenotypic transition connecting genetic and environmental factors to converging endpoints of complicated disorders^26^, metabolomic investigation coupled with biomarker discovery in our study would provide integrative and extensive perspective for better understanding of disease mechanism and causality.

In this study, since the average thyroid hormone levels were within normal range, and most of patients were euthyroid or had mild thyroid function abnormalities, we pursued thyroid autoimmunity associated specific markers rather than thyroid hormone effect. First, we proposed a new biomarker cluster combined with 10 blood metabolites that can discriminate patients with Graves’ disease from the healthy controls. Interestingly, most of the metabolites of the biomarker panel showed the association with a known clinical determinant, TRAb and TSI. The associated metabolites were characterized by carbohydrate metabolism (glucose, mannose, threose, pyruvate, and fumarate) and fatty acid metabolism (pelargonic acid and pentadecanoic acid). Among them were glycerol, an intermediate of both metabolisms that reached the closest univariate statistical criteria between GO and GD (p = 0.28). The potential connectivity was further extended to the intermediates of central carbon metabolism (glucose-6 phosphate, α-ketoglutarate, and pyrophosphate), and nucleotide metabolism (uracil and adenosine-5 monophosphate). Considering that the patients were under euthyroid condition (equivalent level of TSH to the healthy control) but pathological condition, the co-regulated alteration with TSI and TRAb may unveil additional pathological relatedness and new therapeutic approaches beyond TSH regulation.

The subsequent interrogation on the dysregulated metabolites including the biomarkers revealed the potential patho-biochemical linkages. Pathway enrichment analysis indicated overall dysregulation in carbohydrate and amino acid metabolisms. The activation of glycolysis^27^ was accompanied by the integrative activation of PPP^28^ and glutaminolysis^29^. The alteration in the central carbon metabolism may be linked to the hyperactivity in energy metabolism and excessive ROS stress that has been reported in a range of pathogenic immune responses^30–32^. In addition, the increased level in succinate of TCA cycle (p-value 0.06) has been proposed to play a key role in innate immune signaling through enhancement of IL-1b production during inflammation^33^. The enhanced glutaminolysis was reflected in the increased level of α-ketoglutarate and also linked to the accumulation of intermediates in polyamine metabolism essential for T cell activation under GD. As identified in the pathway analysis, the alteration in the range of amino acid metabolisms was a characteristic consequence of the pathophysiology. Excessive levels of leucine and valine may induce excitotoxicity on immune cells and result in the abnormal function of the immune profile^34^.

We further interrogated the unique metabolic dysfunction of Graves’ ophthalmopathy (GO), for which no specific blood biomarker has been developed among patients with Graves’ disease. Two metabolites showed significant differences between the disease groups with or without GO: 1,5-anhydroglucitol and ethanolamine. Note that 1,5-anhydroglucitol and ethanolamine conferred great improvement for discrimination power particularly between GO and the disease group without GO. In addition, we cross-checked the specificity of the biomarker panel against over 40 references (supplementary information file). Among the 12 metabolites proposed in this study, 4 compounds have been reported as the constituents of biomarker from multiple studies on multiple sclerosis (MS) from CSF samples. Less than two metabolites have been reported as biomarkers from other autoimmune diseases (rheumatoid arthritis, systemic lupus erythematosus, Behcet’s disease, neuromyelitis optica spectrum disorders, and coeliac disease), which implied the importance and uniqueness of multi-component biomarker panel as this study^35^.

Next, we sought the unique metabolic signature of the disease group with GO by analyzing orbital fibroblast tissue of patients with the corresponding tissue of healthy controls. Primarily, the tissue-specific metabolic dysregulation was remarked with the intermediates of nucleotide metabolism that was not identified in the blood metabolome. The abnormality in nucleotide metabolism may indicate the higher demands on RNA and DNA synthesis for proliferation under immune cell activation^36^. Likewise, cellular reprogramming of glycolytic pathway under this disease condition implied enhanced bioenergetics, which showed an opposite pattern to the one in the blood. The analysis also determined the coordinated changes between tissue and blood levels in proline, fumarate, and phenylalanine. The aromatic amino acid, phenylalanine contents are closely associated with immune activation and inflammation^37^. The result emphasized the significance of the parallel investigation on biofluid and tissue (organ).

There are some limitations of our study. The samples used in this study were from patients with relatively mild to moderate thyroid function abnormalities due to an avoidable medication for GO prior to surgeries^38^. The medication and a broad spectrum of disease duration may affect the clinical consequences (e.g. decreased disease activity and orbital decompression)^39^, and in turn the disease metabolome observed in blood and tissue although our multiple statistical evaluation proposed that the metabolomic dys-regulation was mainly affected by the disease category rather than by other confounding factors.

In summary, our study revealed the metabolome-wide dysregulation in GD by blood primary metabolite profiles that resulted in the characteristic metabolic linkage, putative biomarker panel discovery, and potential linkage to causative association with important clinical marker, TRAb. In addition, we proposed unique metabolic features of GO that were further elaborated by the comparative analysis of orbital adipose/connective tissues, which may improve the pathological understanding of GO, and suggest potential therapeutic targets.

## Methods

### Patient information and clinical manifestations

We evaluated patients with thyroid disease from October 2014 to January 2016 in Severance Hospital, Seoul, South Korea. Among these patients, patients with Graves’ disease diagnosed by previously known criteria, such as clinical symptoms of hyperthyroidism, in accordance with thyroid function test, and positivity of TRAb were included^40^. The presence of Graves’ ophthalmopathy was diagnosed by ophthalmologists. A total of 48 patient blood samples were obtained, of which 26 patients have Graves’ ophthalmopathy (GO group) and 21 patients have Graves’ disease without Graves’ ophthalmopathy (GD group). Blood samples from 32 healthy controls without clinical evidence of Graves’ disease were also obtained. We collected and reviewed demographic and clinical data, including gender, age, clinical symptoms, thyroid function tests (TFT), thyroid autoantibodies, and thyroid medication history. The severity of ocular manifestation was assessed using clinical activity scores (CAS) of EUGOGO Classification^41^, the modified NOSPECS^42^, and exophthalmometry at the time of blood sampling. This study was approved by and all the method were carried out in accordance with the Institutional Review Board of the Yonsei University College of Medicine (4-2014-0520). All of the patients provided informed consent.

### Thyroid function and antithyroid antibody tests

For thyroid function evaluation, we measured serum concentrations of TSH (normal range, 0.35–4.94 IU/mL), free T4 (normal range, 0.70–1.48 ng/dL), and T3 (normal range, 0.58–1.59 ng/mL) using a microparticle chemiluminescence immunoassay (Abbott Ireland Diagnostics Division, Longford, Ireland). Thyroid status of patients was defined as follows^40,43^: euthyroid included TSH level within the normal range; hypothyroid patients had a low free T4 concentration and elevated TSH levels, whereas patients with subclinical hypothyroidism had elevated TSH with normal free T4; hyperthyroid patients had elevated free T4 with suppressed thyrotropin; and subclinical hyperthyroid was defined as a low thyrotropin with normal free thyroxine.

Thyrotropin receptor antibodies were evaluated by two different methods: M22-TRAb (TRAb) was checked using a third-generation TBII electrochemiluminescence immunoassay (Elecsys/Cobas; Roche Diagnostics, Mannheim, Germany), and Mc4-TSAb (thyroid-stimulating immunoglobulin, TSI) was measured using the Thyretain™ TSI reporter Bio Assay (Diagnostic Hybrids, Inc., Athens, OH, USA). The positivity of antibody tests was defined as TRAb levels higher than 1.75 IU/L or Mc4-TSAb levels higher than the standard value of the sample ratio of 140%.

### Sample preparation

All individuals fasted for 8 h prior to the blood collection. The blood samples were collected in BD Vacutainer tubes containing the chelating agent K_2_ ethylenediaminetetraacetic acid (EDTA), and centrifuged at 2,000 g for 10 min at room temperature. The plasma samples were separated, aliquoted, and stored at −80 °C. Once all samples were collected, the tubes were marked with a unique number to blind them.

### Tissue collection

Orbital adipose/connective tissues were obtained from surgical waste of 5 patients with GO during orbital decompression surgery for severe proptosis (3 men and 2 women; mean age of 53.6 years) (Supplementary Table S1). We also collected normal control fat/connective tissues from 5 individuals without history or clinical evidence of autoimmune thyroid disease or GO during eyelid or orbital surgery for non-inflammatory conditions (3 men and 2 women; mean age of 52.8 years). All 5 patients with GO had well-controlled thyroid function at the time of surgery with or without antithyroid medication. None of the patients with GO was treated with radiation before the orbital surgery. Clinical activity scores of GO patients were less than 4. This study was approved by and all the method were carried out in accordance with the Institutional Review Board of the Yonsei University College of Medicine (4-2014-0292). All of the patients provided informed consent.

### Blood plasma metabolite extraction

The extraction process, derivatization, and mass spectrometric analysis were performed for all samples in randomized order. The extraction procedure, which was conducted for primary metabolite profiling, was as follows: an aliquot of serum samples (50 µL) was extracted with 750 µL of tertiary organic solvent (methanol:isopropanol:water, 3:3:2, v/v/v). The mixtures were shortly vortexed, sonicated for 5 min, and centrifuged for 5 min (13,200 rpm at 4 °C). The supernatant (700 µL) was transferred to a new 1.5-mL tube and dried in a speed vacuum concentrator (SCANVAC, Korea). Dried residual was stored at −80 °C until mass spectrometric analysis.

### Tissue metabolite extraction

The obtained tissue was freeze-dried until analysis. The lyophilized tissue was ground using a single 5 mm i.d. steel ball, followed by the addition of 0.75 mL extraction solvent of methanol:isopropanol:water (3∶3:2) and was vortexed. After a 5-min centrifugation at 16,100 g, 0.70 mL of extracts was collected and concentrated to dryness for further analysis^44^.

### Chemical derivatization

The dried metabolites underwent methoxyamination with 5 µL of 40 mg/mL methoxyamine hydrochloride (Sigma-Aldrich, St. Louis, MO, USA) in pyridine (Thermo, USA) (90 min at 30 °C). The derivative was then reacted with 45 µL of N-methyl-N-trimethylsilyltrifluoroacetamide (MSTFA + 1% TMCS; Thermo, USA) (1 h at 200 rpm and at 37 °C) for trimethylsilylation process. A mixture (2 µL) consisting of 13 fatty acid methyl esters (FAME) was spiked as a retention index marker, which included C8, C9, C10, C12, C14, C16, C18, C20, C22, C24, C26, C28, and C30^45^.

### GC-TOF MS analysis

The injection of derivatized metabolite was controlled by an Agilent 7693 ALS (Agilent Technologies, Wilmington, DE, USA) in splitless mode. Gas chromatography and mass spectrometry were conducted using an Agilent 7890B gas chromatograph (Agilent Technologies) and Leco Pegasus HT time of flight mass spectrometer (LECO, St. Joseph, MI, USA). Gas oven temperature was set to 50 °C for 1 min, increment at 20 °C/min to 330 °C, and hold for 5 min. Transfer line and ion source temperatures were set to 280 °C and 250 °C, respectively. Mass spectra were acquired ranging from 85 to 500 m/z at a scan rate of 17 spectra/sand a detector voltage of 1650 V^20^.

Data pre-processing was done by ChromaTOF software (version 4.5), which included apex mass values, the entire spectrum, signal-to-noise ratio, peak purity, and retention time. Generic text file (.txt) and netCDF file were produced based on raw file (.peg) for peak identification and semi-quantification. The post-process was performed using *Binbase* algorithm consisting of chromatogram validation, primary RI detection, and validation of unique mass^17,46^.

Quantitative value was computed using the peak height of the single unique mass. Missing values that did not pass the primary criteria were imputed by post-matching and replacement using raw data as previously described^12,47^. To check the analytical stability, a mixture of 30 pure reference compounds were analyzed every 10 samples^48^.

### Statistical analysis

Processed data from both GC- and LC-MS analyses were normalized by the sum of identified peaks or selected features of each chromatogram, respectively. Univariate statistical analyses were conducted using the Statistica software version 7.1 (StatSoft, Tulsa, OK, USA). The multivariate statistical analysis (OPSL-DA and O2PLS) and Receiver Operating Characteristic (ROC) analysis were performed using SIMCA 14 (Umetrics AB, Umea, Sweden). OPLS-DA was performed using 7-fold cross-validation and permutation test (N = 100). For validation of ROC analysis, the 95% confidence interval using bootstrapping was done by Biomarker analysis module implemented in MetaboAnalyst 3.0^36,49^.

Pathway enrichment analysis was performed using MetaboAnalyst 3.0. Pathway analysis with metabolite expression data was evaluated for statistical significance using the hypergeometric test and pathway topology was analyzed based on the relative-betweenness centrality. Multivariate analysis of covariance (MANCOVA) based on the Pillai-Bartlett statistics was conducted^15^ using IMB SPSS Statistics (version 22.0, IBM Corp., Armonk, N.Y., USA).

## Electronic supplementary material

Supplementary information

Supplementary information
