# Supplementary material for: Comparative assessment of Graves’ disease and main extrathyroidal manifestation, Graves’ ophthalmopathy, by non-targeted metabolite profiling of blood and orbital tissue
Source: Sci Rep. 2018 Jun 18;8:9262. doi: 10.1038/s41598-018-27600-0 (PMC6006143; doi:10.1038/s41598-018-27600-0)
Supplement: Supplementary file 2 — Supplementary information [file 41598_2018_27600_MOESM2_ESM.pdf]

Comparative assessment of Graves' disease and main extrathyroidal manifestation, Graves' ophthalmopathy, by non-targeted metabolite profiling of blood and orbital tissue

Dong Yoon Ji[1¶], Se Hee Park, MD[2,3¶], Soo Jin Park[1], Kyoung Heon Kim[4], Cheol Ryong Ku, MD, PhD[2,5], Dong Yeob Shin, MD, PhD[2,5], Jin Sook Yoon, MD, PhD[6], Do Yup Lee[1*], and Eun Jig Lee, MD, PhD[2,5*]

[1]The Department of Bio and Fermentation Convergence Technology, BK21 PLUS Program, Kookmin University, Seoul, Republic of Korea

[2]Division of Endocrinology and Metabolism, Department of Internal Medicine, Yonsei University College of Medicine, Seoul, Republic of Korea

[3]Graduate School, Yonsei University College of Medicine, Seoul, Republic of Korea

[4]The Department of Biotechnology, Graduate School, Korea University, Seoul, Republic of Korea

[5]Institute of Endocrine Research, Yonsei University College of Medicine, Seoul, Republic of Korea

[6]Department of Ophthalmology, Yonsei University College of Medicine, Seoul, Republic of Korea

[¶]These authors contributed equally to this work.

[*]Corresponding Authors:

Eun Jig Lee (ejlee423@yuhs.ac)

Division of Endocrinology and Metabolism, Department of Internal Medicine, Yonsei University College of Medicine, Seoul 03722, Republic of Korea

Phone: +82 2 2228 0833, Fax: +82 2 393 6884

Do Yup Lee (rome73@kookmin.ac.kr)

The Department of Bio and Fermentation Convergence Technology, BK21 PLUS program, Kookmin

University, Seoul 02707, Republic of Korea

Phone: +82 2 910 5733, Fax: +82 2 910 5733

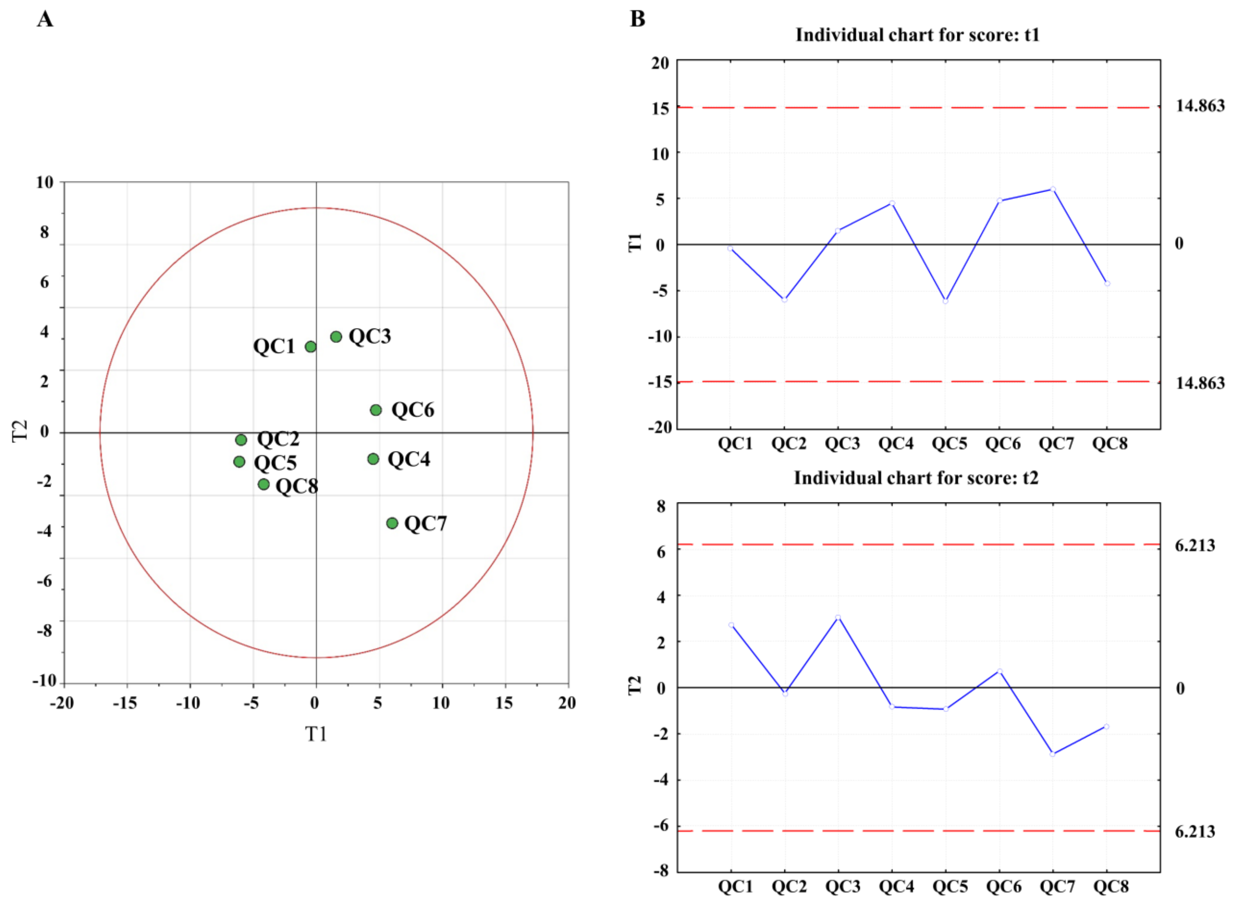

Supplementary Figure S1. Principal component analysis of QC mixture compounds. (A) Score scatter plot of metabolite profiles of 8 QC samples with T1 and T2. X (T1)- and Y(T2)-axis indicate the most and the second most discriminant vectors, respectively. 0.05 as significance level. (B) Line plots (score control chart) with T1 (upper panel) and T2 (lower panel).

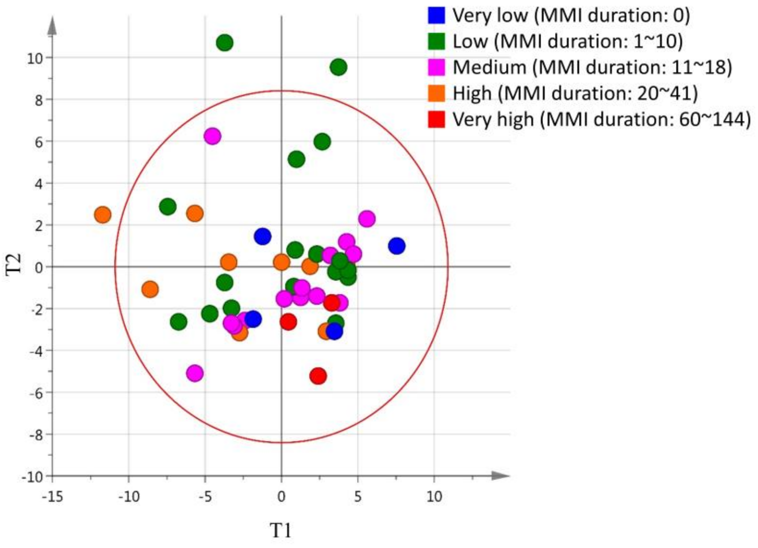

Supplementary Figure S2. Principal component analysis for the potential effects of Methimazole on blood plasma metabolite profiles. Score scatter plot of 47 samples with T1 and T2. X (T1)- and Y(T2)-axis indicate the most and the second most discriminant vectors, respectively. Red circle is the two-dimensional boundary of 47 standard deviation. Color indicates different MMI duration sorted by five periods.

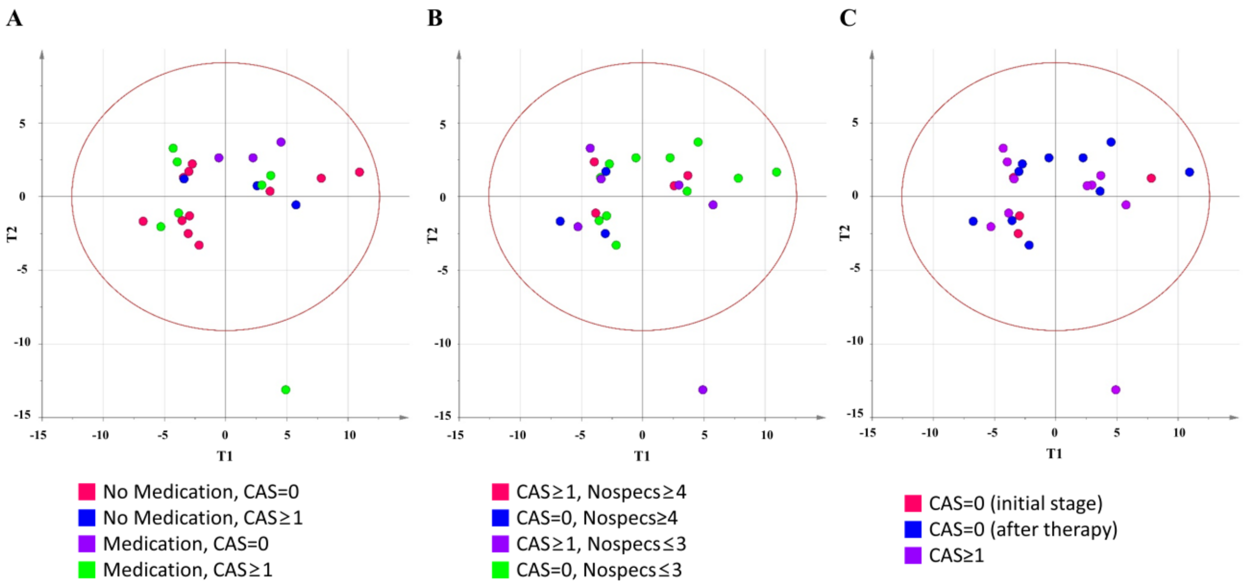

Supplementary Figure S3. Score scatter plots by PCA for four groups categorized by the combination of CAS and steroid medication (A), NOSPECS and CAS (B), and for three groups, CAS=0 (initial stage), CAS=0 (after therapy), and the others (CAS≥1) (C) in the GO group.

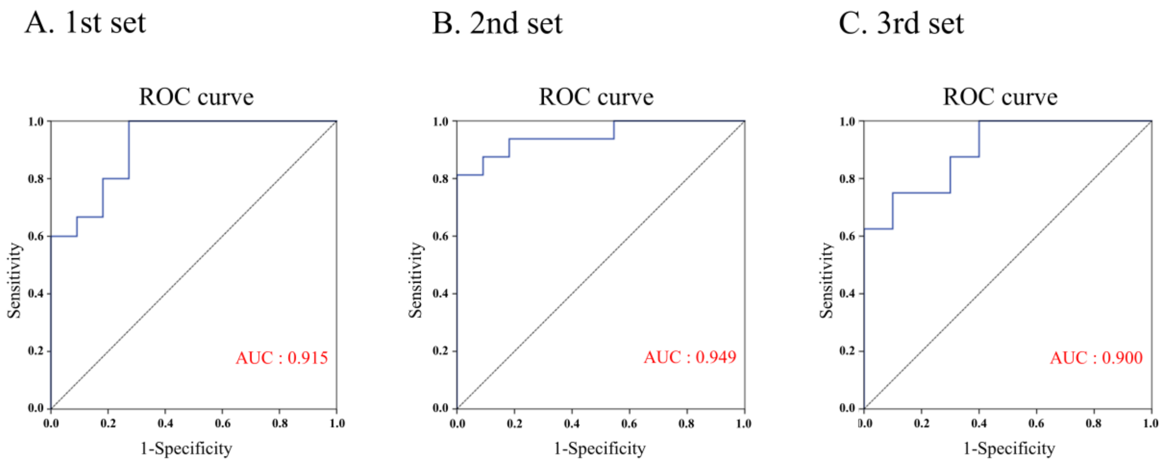

Supplementary Figure S4. Receiver operating characteristic (ROC) analysis of 3 independent test set that thirty percent (26 subjects) of all subjects (79 subjects) are randomly selected and used.

A

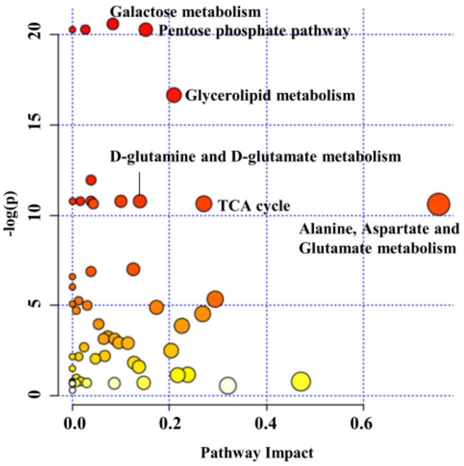

B

Pathway name	Holm p	FDR	Impact
Galactose metabolism	6.59E-08	2.26E-08	0.08333
Starch and sucrose metabolism	8.83E-08	2.26E-08	0.02678
Pentose phosphate pathway	8.83E-08	2.26E-08	0.15109
Amino sugar and nucleotide sugar metabolism	8.83E-08	2.26E-08	0
Glycerolipid metabolism	3.15E-06	6.76E-07	0.20907
Fructose and mannose metabolism	3.44E-04	6.28E-05	0.03835
Ascorbate and aldarate metabolism	1.08E-03	1.03E-04	0.01617
Vitamin B6 metabolism	1.08E-03	1.03E-04	0.03828
Histidine metabolism	1.08E-03	1.03E-04	0.00051
D-Glutamine and D-glutamate metabolism	1.08E-03	1.03E-04	0.13904
Lysine biosynthesis	1.08E-03	1.03E-04	0.09993
Citrate cycle (TCA cycle)	1.13E-03	1.03E-04	0.27058
Glyoxylate and dicarboxylate metabolism	1.13E-03	1.03E-04	0.04303
Alanine, aspartate and glutamate metabolism	1.13E-03	1.03E-04	0.75404

Supplementary Figure S5. Pathway enrichment analysis of plasma metabolite profiles of the Graves' disease group compared to the healthy control. The analysis is conducted on all continuous variables (metabolites) with the relative concentrations without pre-determined statistical cutoff. (A) Pathway impact values, estimated from the number of connection of a node (a metabolite), are plotted against X-axis. The values are computed by number of shortest routes passing through the node (betweenness centrality). The higher score suggests that the metabolite is located in topologically and putatively biochemically important position. P-values are plotted against Y-axis, which presents the significant levels of pathway alteration cumulatively computed by the significance of metabolite changes in a pathway. For visual clarification, the pathway importance and the statistical significance are proportional to node radius and color respectively. (B) The list of metabolic pathways with significant difference the GD group compared to healthy controls. The statistical significance is

evaluated and presented by adjusted p values (Holm-Bonferroni p values) and false discovery rate (FDR).

A

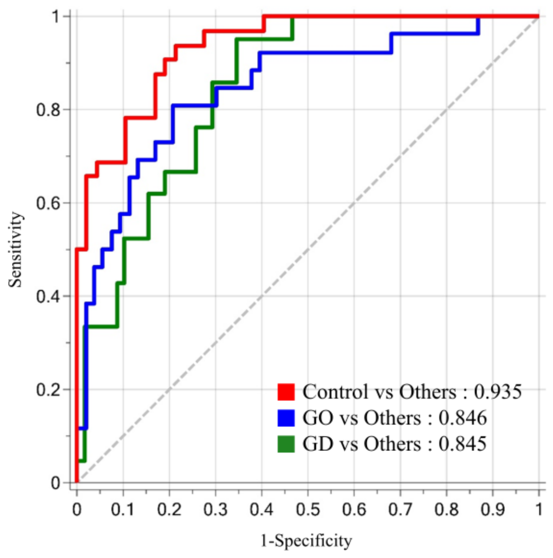

B

Metabolite panel
Glucose
Pelargonic acid
2-(4-hydroxyphenyl)ethanol
Gluconic acid lactone
Glycerol
Pentadecanoic acid
Mannose
Threose
Fumaric acid
Pyruvate
1,5-anhydroglucitol
Proline

Supplementary Figure S6. (A) Receiver operating characteristic (ROC) analysis of multiple metabolite panels for discriminating the three different groups (GO, GD, and healthy control). (B) The list of metabolite panel is provided in the right panel.

Supplementary Table S1. Clinical characteristics of the patients with Graves' ophthalmopathy included in the tissue analysis

Case	Age/Sex	Duration of GO, m	Smoking	Previous treatment	CAS	Proptosis Rt/Lt, mm	Surgical treatment
1	36/F	36	Yes	GC	1/7	21/20	Decompression
2	51/M	12	Yes	GC	3/7	21/23.5	Decompression
3	59/M	192	Yes	GC	2/7	22/21	Decompression
4	61/M	4	Yes	GC	3/7	21/21	Decompression
5	61/F	24	No	GC	3/7	20.5/21	Decompression

The range of CAS was from 0 to 7. GO, Graves' ophthalmopathy; CAS, clinical activity score, GC, glucocorticoid
